# Retracted: Psorinum Therapy in Treating Stomach, Gall Bladder, Pancreatic, and Liver Cancers: A Prospective Clinical Study

**DOI:** 10.1155/2018/6803672

**Published:** 2018-02-26

**Authors:** Evidence-Based Complementary and Alternative Medicine

Evidence-Based Complementary and Alternative Medicine has retracted the article titled “Psorinum Therapy in Treating Stomach, Gall Bladder, Pancreatic, and Liver Cancers: A Prospective Clinical Study” [1] due to concerns about the ethics, authorship, quality of reporting, and misleading conclusions.

Aradeep and Ashim Chatterjee own and manage the Critical Cancer Management Research Centre and Clinic (CCMRCC), the private clinic to which they are affiliated. The methods state “The study protocol was approved by the Institutional Review Board (IRB approval Number: 2001–05) of the CCMRCC” in 2001, but a 2014 review of Psorinum therapy said CCMRCC was founded in 2008 [2]. The study states “The participants received the drug Psorinum along with allopathic and homeopathic supportive treatments without trying conventional or any other investigational cancer treatments”; withholding conventional cancer treatment raises ethical concerns.

We asked the authors and their institutions for documentation of the ethics approval, the study protocol, and a blank copy of the informed consent form. However, the corresponding author, Aradeep Chatterjee, was reported to have been arrested in June 2017 for allegedly practising medicine without the correct qualifications and his co-author and father Ashim Chatterjee was reported to have been arrested in August; the Chatterjees and their legal representative did not respond to our queries. The co-authors Syamsundar Mandal, Sudin Bhattacharya, and Bishnu Mukhopadhyay said they did not agree to be authors of the article and were not aware of its submission; co-author Jaydip Biswas did not respond.

A member of the editorial board noted that although the discussion stated that “The limitation of this study is that it did not have any placebo or treatment control arm; therefore, it cannot be concluded that Psorinum Therapy is effective in improving the survival and the quality of life of the participants due to the academic rigours of the scientific clinical trials”, the abstract was misleading because it implied Psorinum therapy is effective in cancer treatment. The study design was described as a “prospective observational clinical trial”, but it cannot have been both observational and a clinical trial.
